# Genomic imprinting of *IGF2 *in marsupials is methylation dependent

**DOI:** 10.1186/1471-2164-9-205

**Published:** 2008-05-02

**Authors:** Betty R Lawton, Benjamin R Carone, Craig J Obergfell, Gianni C Ferreri, Christina M Gondolphi, John L VandeBerg, Ikhide Imumorin, Rachel J O'Neill, Michael J O'Neill

**Affiliations:** 1 Department of Molecular and Cell Biology, University of Connecticut, 354 Mansfield Rd., Storrs, CT 06269, USA; 2 Southwest Foundation for Biomedical Research, P.O. box 760549, San Antonio, TX 78245-0549, USA; 3 Dept. of Biology, Spelman College, Atlanta, GA 30314, USA

## Abstract

**Background-:**

Parent-specific methylation of specific CpG residues is critical to imprinting in eutherian mammals, but its importance to imprinting in marsupials and, thus, the evolutionary origins of the imprinting mechanism have been the subject of controversy. This has been particularly true for the imprinted *Insulin-like Growth Factor II *(*IGF2*), a key regulator of embryonic growth in vertebrates and a focal point of the selective forces leading to genomic imprinting. The presence of the essential imprinting effector, *DNMT3L*, in marsupial genomes and the demonstration of a differentially methylated region (DMR) in the retrotransposon-derived imprinted gene, *PEG10*, in tammar wallaby argue for a role for methylation in imprinting, but several studies have found no evidence of parent-specific methylation at other imprinted loci in marsupials.

**Results-:**

We performed the most extensive search to date for allele-specific patterns of CpG methylation within CpG isochores or CpG enriched segments across a 22 kilobase region surrounding the *IGF2 *gene in the South American opossum *Monodelphis domestica*. We identified a previously unknown 5'-untranslated exon for opossum *IGF2*, which is flanked by sequences defining a putative neonatal promoter, a DMR and an active Matrix Attachment Region (MAR). Demethylation of this DMR in opossum neonatal fibroblasts results in abherrant biallelic expression of *IGF2*.

**Conclusion-:**

The demonstration of a DMR and an active MAR in the 5' flank of opossum *IGF2 *mirrors the regulatory features of the 5' flank of *Igf2 *in mice. However, demethylation induced activation of the maternal allele of *IGF2 *in opossum differs from the demethylation induced repression of the paternal *Igf2 *allele in mice. While it can now be concluded that parent-specific DNA methylation is an epigentic mark common to Marsupialia and Eutheria, the molecular mechanisms of transcriptional silencing at imprinted loci have clearly evolved along independent trajectories.

## Background

*Monodelphis domestica *is a South American opossum of the family Didelphidae, belonging to the mammalian infraclass Marsupialia, which last shared a common ancestor with Eutheria approximately 145 million years ago [1]. Marsupials possess a non-invasive yolk sac placenta that allows for maternal-fetal nutrient exchange following implantation [2]. Thus, while marsupials differ from eutherians in several embryological features, notably: the absence of both a morula stage and an inner cell mass during development [3], they can, nonetheless, be considered "placental" mammals.

Marsupials also share with eutherians genomic imprinting, resulting in parent-specific gene expression (PSGE) at several loci in embryonic and placental development. Examination of allele-specific expression patterns of *IGF2 *in *M. domestica *and the chicken *Gallus gallus *showed paternal monoallelic expression in opossum but biallelic expression in birds [4], supporting the Kinship Theory of genomic imprinting [5]. PSGE has since been demonstrated for the genes *IGF2R*, *PEG1/MEST*, and *PEG10 *in marsupials, but thus far PSGE has not been found in birds or egg-laying mammals, the monotremes [6-8].

Imprinted expression of most genes in eutherians is dependent upon parent-specific differential methylation of specific CpG residues within imprinted loci. The indispensability of CpG methylation to the maintenance of genomic imprints in eutherians was first demonstrated by the loss of imprinting in mice carrying targeted mutations of the maintenance DNA methyltransferase gene, *Dnmt1 *[9]. Furthermore, mice carrying targeted mutations of the gene, *Dnmt3L*, demonstrated the necessity of this DNA methyltransferase-like gene to the establishment of genomic imprints in the female germline. A *Dnmt3L *ortholog has been identified in marsupials [10], but the importance of methylation to imprinting in marsupials has remained controversial since several studies have failed to identify differentially methylated regions (DMRs) associated with imprinted loci in marsupials [6,8,11]. However, the recent discovery of a DMR associated with the *PEG10 *gene in the tammar wallaby is the first demonstration that differential methylation is critical for imprinted expression of a gene in marsupials [12]. *PEG10 *is a gene derived from the retrotransposon *sushi ichi *leading these authors to hypothesize that mammalian genomic imprinting evolved from a host defense mechanism to silence selfish mobile elements [12].

In eutherians, imprinting of *IGF2 *depends upon allele-specific interaction of DMRs within and near the *IGF2 *transcription unit [13,14]. In mouse, the DMRs include DMR1, a paternally methylated repressor element adjacent to the fetal-specific promoter [15], DMR2, a paternally methylated element in the last protein-coding exon of *Igf2 *[16], and the H19-DMD imprinting control region (ICR) located 90 kb downstream of *Igf2 *that binds the insulator CTCF on the unmethylated maternal allele [15,17]. Both DMR1 and DMR2 associate with MARs that allow for long-range interactions with the ICR [13,14,18]. The interaction of DMR1 with the ICR on the maternal allele generates a chromatin loop enclosing the *Igf2 *promoter region and effectively preventing transcription of the gene from this allele. The paternal allele, however, features an interaction of DMR2 with the ICR, which places the *Igf2 *promoter outside of the chromatin loop where transcription activating factors are presumably available.

To date, no DMRs have been reported at marsupial *IGF2 *loci, nor has a marsupial ortholog of *H19*. To examine the mechanism of genomic imprinting of the *IGF2 *locus in marsupials we tested the necessity of CpG methylation for mono-allelic expression of *IGF2*, performed 5' RACE (rapid amplification of cDNA ends) to identify the site of transcription initiation and promoter region of *IGF2 *and assayed a 22 kilobase region surrounding this gene for DMRs and MARs in neonatal tissues of *Monodelphis domestica*.

## Results

### 5-azacytidine treatment of neonatal fibroblasts

**Figure 1:**
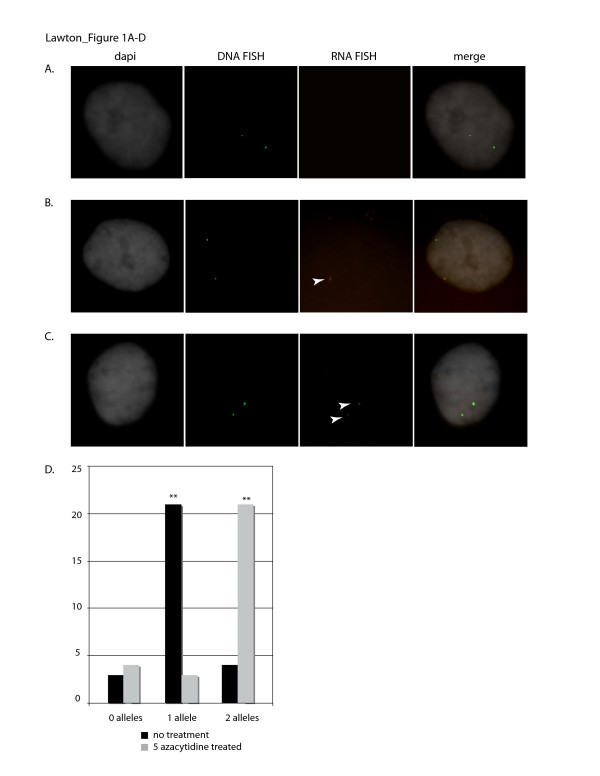
**Biallelic expression of *IGF2 *in *M. domestica *is dependent on CpG methylation.** A-C) RNA fluorescence *in situ *hybridization (FISH) detecting nascent transcripts of *IGF2 *(red) and DNA FISH detecting DNA copies of *IGF2 *(green) to interphase cells (counterstained with dapi, black and white). Individual probe images for dapi, DNA FISH, RNA FISH and the merge image are shown from left to right. A) FISH on control (no treatment) cells showing no *IGF2 *expression. B) FISH on control (no treatment) cells showing monoallelic *IGF2 *expression (RNA transcript is indicated with an arrowhead). C) FISH on 5-azacytidine treated cells showing biallelic expression (RNA transcripts are indicated with an arrowhead). D) Total cell counts/well of 0, 1 or 2 RNA FISH signals in experiment and control micrographs as designated.

Day 0 neonate fibroblast cells were treated with 5-azacytidine followed by RNA/DNA fluorescence *in situ *hybridization (FISH) to ascertain the affect of CpG hypomethylation on the allelic expression profile of *IGF2 *in *M. domestica*. Both treated and control cells were subjected to RNA FISH to determine mono-vs biallelic expression status, followed by DNA FISH as an internal hyrbidization control. Only those cells containing two DNA FISH signals were scored for RNA FISH (Figure 1A–C). Untreated neonate cells showed zero RNA signals in 11.1% of cells (Figure 1A), one RNA signal in 77.7% of cells (Figure 1B), and two RNA signals in 11.1% of cells, confirming the imprinted status of *IGF2 *(Figure 1D). Cells treated with 5-azacytidine showed a significant (p < 0.001) shift to biallelic expression, with 14.3% of cells containing no RNA signal, 10.7% containing one RNA signal and 75% of cells containing 2 RNA FISH signals (Figure 1C,D). We have previously shown that only the paternal allele of *IGF2 *is transcriptionally active in neonatal opossums [4]. The detection of two RNA FISH signals in treated neonatal fibroblasts, therefore, indicates that the normally silent maternal allele has been activated by the loss of cytosine methylation.

### IGF2 transcription unit analysis

**Figure 2:**
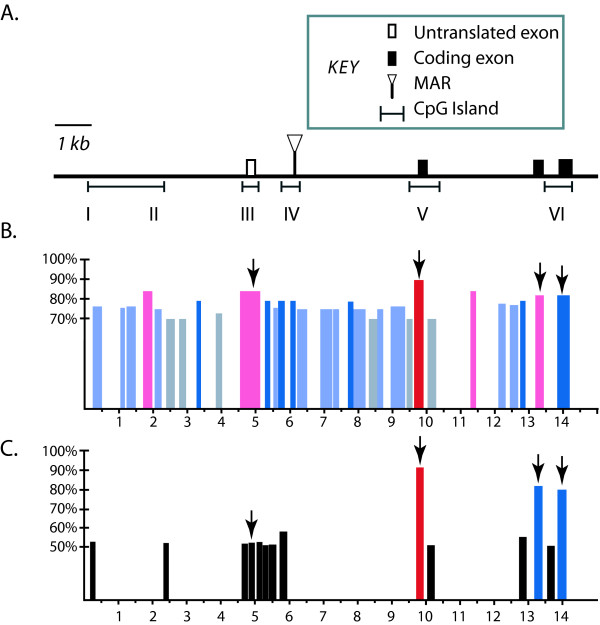
**Characterization of the transcription unit for *IGF2 *in *Monodelphis domestica*.** A) Genomic map of the *M. domestica IGF2 *gene. The scale bar is indicated and the key to the annotation, which includes coding exons, the 5' noncoding exon, the MAR predicted by MAR-Wiz and CpG islands predicted by CpG Plot, is shown below. B) & C) Evolutionary conserved segments within the *M. domestica IGF2 *locus. VISTA pairwise comparisons showing conserved segments between *M. domestica *and B) *Macropus eugenii*, C) *Homo sapiens*. Percent homology is indicated on the Y-axis and the position and size of regions of high homology are indicated on the X-axis in kilobases according to the reference sequence (*M. domestica*). Arrows denote the four exons in *M. domestica*. Black bars indicate >50% identity, light blue bars indicate >75% identity, dark blue bars indicate >80% identity, pink bars indicate >85% identity, and red bars indicate >90% identity.

Slightly greater than 22 kilobases of DNA sequence encompassing the *M. domestica IGF2 *gene was assembled from sequencing of a 14 kb lambda clone and from subcloning and sequencing regions of a BAC clone (223O16, pCC1BAC) [19]. A northern blot of RNA extracted from whole neonate tissue was hybridized to a cDNA probe encompassing the protein coding portion of the *M. domestica IGF2 *gene. A single transcript of approximately 5 kb was detected (data not shown). To determine the 5' extent of the transcript and identify the promoter for the gene we performed 5' RACE using oligonucleotide primers seated in the first protein coding exon. 5' RACE of neonatal RNA generated a single product that revealed a 95 base pair non-coding exon situated ~5 kilobases upstream of the start codon in the first protein coding exon of *IGF2 *(Figure 2A). In mouse and human, multiple transcripts for *IGF2 *have been detected with specific spatiotemporal expression profiles that result from differential promoter usage and alternative splicing of noncoding exons upstream of the three conserved protein coding exons. The single band detected by northern analysis and the single 5' RACE product, both from whole neonatal RNA, suggest that opossum neonates express only one *IGF2 *transcript isotype from one promoter. The 5' flanking region of the noncoding exon identified by 5' RACE was analyzed by PROSCAN [20] and was predicted with high confidence to be a promoter (promoter score = 73.63).

We next determined if features of the *M. domestica IGF2 *locus, including the region encompassing the 5' noncoding exon and the predicted promoter, show conservation among marsupial clades and between marsupials and eutherians. Pairwise alignments using a 100 base pair sliding window were performed in VISTA [21] utilizing the genomic sequences of *IGF2 *from *M. domestica*, *Macropus eugenii *(tammar wallaby) and *Homo sapiens *(Figure 2B,C). Conservation was extremely high (>80%) between all three species within the three protein coding exons. In addition, a segment of approximately 600 base pairs, encompassing the 5' noncoding exon and upstream flanking sequence showed >85% identity between opossum and tammar wallaby and >50% identity between opossum and human. Didelphids (including *Monodelphis *and *Didelphis*) and Macropodids (including *Macropus*) last shared a common ancestor ~85 MYA, while estimates for the most recent common ancestor for human and *M. domestica *are ~145 MYA [1]. Given these deep phylogenetic branches, sequences containing high homology between these disparate species may be indicative of functional conservation and represent potential targets for further epigenetic analyses.

### CpG Methylation Analyses

**Figure 3:**
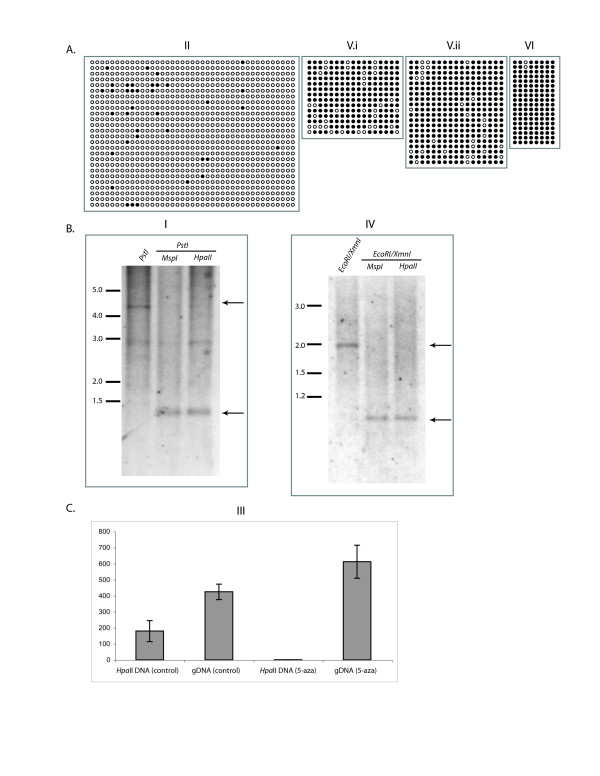
**Methylation analysis of the CpG islands across the *M. domestica IGF2 *locus.** Each Roman numeral corresponds to the CpG island indicated in Figure 2A. A) Bisulfite sequencing results for CpG islands II, V and VI. Filled circles represent methylated cytosines, and unfilled circles unmethylated cytosines. B) Methylation sensitive Southern analysis of CpG islands I and IV. The enzyme used to identify non-methylated CpGs are indicated at the top of the corresponding lane, the ladder sizes are shown to the left, and arrows to the right indicate the hybridizing bands for *IGF2*. C) Real time genomic PCR assay for CpG island III. Levels of PCR product from *HpaII *digested DNA, non-digested genomic DNA (control), *HpaII *digested 5-azacytidine treated DNA and non-digested, *5-*azacytidine treated genomic DNA are shown. Error bars represent 99.9% confidence limits.

Several CpG isochores and CpG enriched regions were predicted within the assembled sequence contig using CpG Plot [22]. A CpG enriched region was found upstream and within the third coding exon (region VI, Figure 2A) corresponding to DMR2 in mice, which is paternally methylated and known to be important in maintaining high levels of transcription of *Igf2 *from the paternal allele [16]. Bisulphite sequencing of this CpG enriched region encompassing 750 base pairs in *M. domestica *revealed no differential methylation (Figure 3A).

No sequence cognates of mouse *Igf2 *DMR0 or DMR1 were identified. Nevertheless, bisulfite sequencing and methylation-sensitive Southern analyses were performed on the CpG enriched regions found upstream of the first exon (regions I and II, Figure 3A,B), within the first intron (region IV, Figure 3B) and flanking the second exon (region V, Figure 3A). Like region VI, region V is fully methylated on both alleles, while regions I, II and IV are unmethylated on both alleles.

Region III includes the first exon and approximately 500 base pairs of its immediate 5' flank. Apart from the predicted promoter, this region is composed of extremely low complexity DNA sequence: the upstream half of which is comprised of interspersed strings of G's or A's two to six bases in length, while the downstream half is comprised of similarly short strings of C's and T's. The character of the sequence in this region precludes both the ability to design primers for bisulphite sequencing, which requires high complexity sequence to compensate for the conversion of C's to U's by deamination, as well as the utilization of methylation sensitive restriction enzymes for Southern analysis to detect differential CpG methylation. Therefore, we designed a methylation-sensitive restriction digest/quantitative real-time PCR assay to examine select CpG residues in the vicinity of the predicted promoter. The quantitative real-time assay detects 50% methylation (99.9% confidence limits) within Region III (Figure 3C). Furthermore, the 50% methylation of Region III is lost upon 5-azacytidine treatment of cells. Sequence polymorphisms that might distinguish parental copies of Region III were not found among the available stocks of *M. domestica*, therefore it is formally possible that the CpGs in this region are randomly methylated. However, the precise 50% methylation (within 0.1% error) of Region III despite full methylation or unmethylation of CpGs in surrounding regions, and the activation of the maternal allele of *IGF2 *concurrent with the loss of methylation of Region III in 5-azacytidine treated neonatal fibroblasts, strongly suggest that Region III comprises an allele-specific DMR essential to PSGE of *IGF2 *in opossums.

### Matrix Attachment Regions

**Figure 4:**
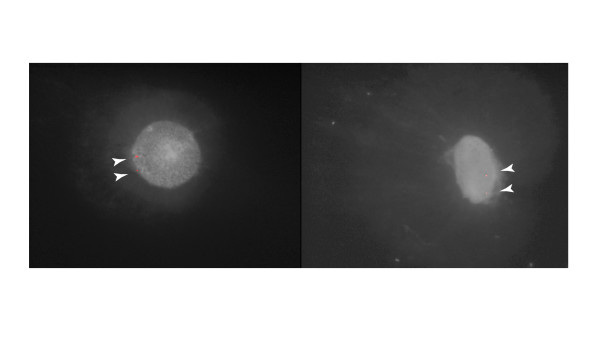
**MAR-predicted sequence within *IGF2 *is found in the nuclear matrix.** Fluorescence *in situ *hybridization of a 5 kb *IGF2 *subclone (red) encompassing sequence predicted to contain a MAR on nuclear halo preparations (counterstained with dapi and shown in black and white). The positive hybridization signals are found only in the nuclear matrix and are indicated with arrowheads. Two different cells are shown (left and right).

When sequence obtained from the 5' region of the *M. domestica IGF2 *gene was input into the bioinformatics program MAR-Wiz [23], a region within the first intron just downstream of the 5' noncoding exon was predicted to be a Matrix Attachment Region (92% average strength) (Figure 2A). FISH using a 5 kilobase probe containing the predicted MAR sequence showed two signals exclusively in the nuclear matrix rather than in the loop region of nuclear halos (Figure 4), confirming the presence of an active MAR in the 5' region of this gene in opossum.

## Discussion

Research published in the aftermath of the discovery of genomic imprinting in marsupials found no evidence of parent-specific CpG methylation at imprinted loci, the hallmark of genomic imprinting in eutherians [6,8,11]. As a consequence, there has been speculation that there has been evolutionary divergence of imprinting mechanisms between eutherians and marsupials, with differential CpG methylation proposed as a eutherian innovation [24]. However, the observation that marsupials carry the genes for both *DNMT3L *[10] and *DNMT1o *[25], both key factors in establishing and/or maintaining genomic imprints in eutherians [26,27], suggested a role for CpG methylation in marsupial imprinting. Furthermore, recent investigation of the regulation of tammar wallaby *PEG10 *suggested that differential methylation in response to a retrotransposition in the common ancestor to marsupials and eutherians may constitute the mechanistic origin of genomic imprinting in mammals [12]. Interesting in this regard, the DMR in the opossum *IGF2 *promoter shows no evidence of having been derived from a mobile element.

Prior efforts to find differential methylation at the *IGF2 *locus in opossum has likely been hampered by the fact that this locus is highly enriched in low complexity polynucleotide repeats. This has made demarcation and localization of exons and assembly of long contiguous sequence at the *M. domestica IGF2 *locus a difficult task. Nevertheless, we have now shown that imprinted expression of *IGF2 *in opossum is dependent on CpG methylation and that sequences adjacent to the putative promoter of this gene appear to be differentially methylated in a parent-specific manner. Further comparative studies between opossum and the tammar wallaby, including promoter-reporter assays and allele-specific methylation studies, may reveal conservation of these features among Marsupialia.

The DMR at the promoter of *IGF2 *in opossum may be analogous to the DMR1 just upstream of *Igf2 *promoter P1 in mouse. Imprinted expression of *Igf2 *in mesodermal tissues of the mouse embryo depends on the interaction of DMR1 with the *H19 *DMD located ~45 kilobases downstream of *Igf2 *and 2–4 kilobases upstream of the *H19 *gene [13,28]. The absence of methylation on the maternal copy of the *H19 *DMD allows for the binding of CTCF and assembly of a chromatin insulator at that site [17]. Assembly of this insulator is indispensable to the transcriptional silencing of the maternal *IGF2 *allele. Mouse embryos in which the gene for the maintenance DNA methyltransferase, *Dnmt1*, has been knocked out by gene targeting express *H19 *from both parental alleles while silencing both alleles of *Igf2 *[29]. The activation of the maternal allele of *IGF2 *in neonatal opossum cells in which differential methylation of the Region III DMR has been abolished by 5-azacytidine treatment suggests an important divergence between marsupials and eutherians in the epigenetic regulation of *IGF2 *imprinting. To date, no report of marsupial orthologs of *H19 *have been published.

Analysis of the distribution and function of matrix attachment regions (MARs) at the opossum *IGF2 *locus also shows striking contrasts to the mouse *Igf2 *locus. MAR-wiz analysis of 22 kilobases encompassing the *M. domestica IGF2 *transcription unit only identified one MAR with strong statistical support. Three MARs (MAR1, 2, 3) have been identified in mouse and human that are conserved in location with respect to *IGF2 *intron/exon structure. Two of these, MAR2 and MAR3, show allele-specific matrix attachment in mouse that varies by tissue and developmental stage [14,30]. These MARs allow for long-range interactions between the *Igf2 *DMRs and the *H19 *DMD, creating a chromatin conformation that places the *Igf2 *promoter inside DNA loops on the maternal allele to prevent transcription, or forming a transcriptional complex on the paternal allele at the nuclear matrix [13,14].The VISTA alignment of opossum and human *IGF2 *suggests that the opossum MAR may play an analogous role to MAR1, which in mouse, like opossum, does not show parental-allele specific matrix attachment.

## Conclusion

The critical role of *IGF2 *signaling during vertebrate development, the imprinting of *IGF2 *and several genes modulating its activity in placental mammals [31], and the demonstration of sustained Darwinian selection on *IGF2 *in placental fishes [32], places this gene at the crux of the parent-offspring conflict that accompanies the adaptation to placental reproduction. It is most parsimonious to assume that genomic imprinting of *IGF2 *evolved in the common ancestor of Eutheria and Marsupialia, and the demonstration that imprinting relies on allele-specific cytosine methylation in both supports this notion. However, we have shown that the epigenetic signals and chromatin conformation that govern imprinting at this locus in opossum are dramatically different than that in mouse, suggesting that the mechanism of transcriptional silencing of the maternal allele may have evolved independently in these two lineages.

## Methods

### Cell Culture

Primary cultures of fibroblast cells were established from (Day 0) neonates.

Neonate tissues were treated with 500 units Collagenase type 3 (Worthington Biochemical), in 35 mm Petri dishes containing complete marsupial media [33]. Tissues were excised with a scalpel blade and incubated 18–24 hours at 37°C. Disaggregated cells were then transferred to 15 mL Falcon tubes and centrifuged to collect cells and rinsed with sterile PBS to remove remaining Collagenase. Cells were further disaggregated with a 1 mL syringe capped with a 21G needle, transferred to flasks (25 cm^2^) and grown to confluency at 35°C in complete marsupial media. Optimal 5-azacytidine treatment recovery time was determined empirically. Four flasks (25 cm^2^) with cells at ~60% confluency were treated with 20 uM 5-azacytidine in complete marsupial media for 24 hours followed by 24, 48, 72 and 96 hours of recovery, respectively. DNA was isolated from each flask and 1 μg was digested with *HpaII *overnight. The recovery time of 48 hours contained cells showing the greatest decrease in CpG methylation and was subsequently chosen for further analyses (data not shown).

### RNA FISH

*Igf2 *probe was derived from 5 kb of intronic sequence 5' of the first noncoding exon. The subclone was generated from *M. domestica IGF2 *BAC DNA clone # 223O16, pCC1BAC [34] from the VMRC18 library (CHORI) digested with *EcoR I *and *Bgl II *and ligated into pBluescript^® ^KS+ (Stratagene). Plasmid DNA (1 μg) was labeled with dig-11-dUTP using the Dig High Prime (Roche) Kit for RNA FISH and with biotin-16-dUTP with the Biotin High Prime (Roche) Kit for DNA FISH, both according to manufacturer's instructions. Probe DNA was precipitated with 10 μg salmon sperm DNA, 10 μg tRNA, 20 μg sonicated *M. domestica *genomic DNA and 2 volumes -20°C ethanol for 30 minutes at -80°C. Following centrifugation for 20 minutes, hybridization cocktail DNA was resuspended in 17 μl of 100% deionized formamide and 17 μl 2 X hybridization mix [35]. Hybridization cocktail DNA was denatured at 70°C for 10 minutes and incubated at 37°C for 30 minutes to allow blocking of repeat sequences in the probe DNA.

Day 0 neonate fibroblast cells were plated on a Dual Chamber slide in DMEM media supplemented with 10% FBS and non-essential amino acids overnight at 35°C. Once the cells were at ~60% confluency, one of the two wells for each slide was treated with 5-azacytidine (20 uM) for 24 hrs, while the control well was treated with media alone. After 24 hours of treatment, both wells were rinsed with PBS and treated with media for 48 hours of recovery at 35°C. Following recovery, slides were processed in cytoskeletal buffer for 2.5 minutes, followed by formaldehyde fixation and 2 × 70% ethanol rinses according to [36].

Following overnight hybridization at 37°C under a 22 × 50 mm coverslip across both the treated and control wells, slides were washed in 50%formamide/2 × SSC at 45°C for 3 washes of 5 minutes each followed by 1 × SSC (prewarmed to 60°C) at 45°C for 3 washes of 5 minutes each. Detection using anti-dig-rhodamine was performed according to [37]. Cells were counterstained with DAPI, mounted in antifade and viewed on a Leica DM6000B equipped with a DFC350FX CCD camera and analyzed on a CW4000 Cytogenetics Image Analysis Workstation. Once positive hybridization signals were visually confirmed, the slide was rinsed in PBS to remove the coverslip and antifade solution and was subsequently processed for DNA FISH. The slide was treated with RNAse A (0.1 mg/mL RNAse A in 2 × SSC). Following chromosome denaturation in 70%formamide/2 × SSC at 70°C for 2 minutes the slide was dehydrated in ice cold 70%, 90% and 100% ethanol.

DNA FISH probe (1 μg biotin labeled DNA) was precipitated according to RNA FISH (above). The DNA FISH hybridization cocktail was denatured and pre-annealed and applied to the slide as above. FISH washes were performed as above followed by detection with anti-biotin FITC Avidin Fluorescein (Vector Laboratories).

Hybridization signals were scored for a minimum of 25 cells per well [38]. Only cells containing two positive DNA hybridization signals were scored for use in statistical analyses. Chi tests were performed to determine whether the observed peak signal distribution of hybridization signals within the treated well was significantly different than the peak signal distribution of the control sample.

### Isolation and sequencing of M. domestica IGF2

A *Monodelphis domestica *genomic library constructed in Lamda FIX^®^II/*Xho*I (Stratagene, according to the manufacturer's protocol) was screened using a probe generated from amplification of genomic DNA from a male *M. domestica *liver with primers designed from an alignment of the 3' untranslated region (UTR) of several species [4]. Subclones were generated by restriction digests with *BamH I*, *EcoR I*, *Not I*, and *Sac II *and ligation into pBluescript^® ^II KS+ vector (Stratagene). Additional sequence was obtained by screening the VMRC-18 *M. domestica *genomic library ordered from BACPAC Resources (Children's Hospital Oakland Research Institute, Oakland, CA) using the same 3' UTR probe. BAC DNA was prepared using a QIAGEN™ Plasmid Midi Kit following manufacturer's instructions. Sequencing was accomplished by primer walking using BigDye Chemistry (Applied Biosystems), according to the manufacture's protocol. Regions of repeats or secondary structure were subcloned after *Pst I *digestion into pBluescript^® ^KS+, then sequenced using 400 ng DNA, 2.3 μL of 5× buffer, 5% DMSO (final concentration), 1 M Betaine (final concentration), 2 μL BigDye Terminator v3.1, dGTP BigDye Terminator v3.0 (Applied Biosystems), 3.2 picomoles primer, and MilliQ water to 20 μL. The reaction was run for 30 cycles at 95°C for 15 sec., 50°C for 5 sec., and 60°C for 2 min.

### Detection of noncoding exons

The 5' untranslated exon sequence was captured by RACE using the BD SMART™ RACE cDNA Amplification Kit. The procedure was done as described by the manufacturer using 1 ug of total RNA, a reverse gene specific primer (5': GACGCTTGGCCTCTCTGAT) in the primary PCR, and a reverse nested gene specific primer (5: CGGGGAATCTGGGGAAGTTGTCC). The resulting amplicon was cloned into the pCR^® ^II Topo^® ^vector and sequenced.

### Sequence analyses

The full 22 kb from *M. domestica IGF2 *and *Macropus eugenii *(BAC MEKBa-346C2, gi: CR925759) were analyzed in VISTA [21] using a 100 bp sliding window. Sequence identity greater than 70% over 100 bp was scored. The homologous segment of human *IGF2 *(UCSC Genome Browser: hsa chr11:2107400-2125903) was used in pairwise VISTA alignments to identify conserved portions within a 100 bp sliding window with greater than 50% identity.

### Assessment of differential methylation

CpG islands were predicted by the program CpG Plot with input analyzed using an observed/expected ratio of 0.6 and a window length of 200 bp [22]. Either bisulfite sequencing or southern analysis was conducted on CpG islands to evaluate the presence of differential methylation. Bisulfite sequencing was performed on neonate liver DNA. Briefly, 4 μg of DNA extracted from a Day 0 neonate was treated with bisulfite reagents using the EZ DNA Methylation Kit (Zymo Research) as per manufacturer's instructions. Four microliters of treated DNA was then amplified using 100 ng forward and reverse primers designed in MethPrimer [34] (see Additional file 1) in a 35 cycle protocol consisting of 94°C for 1 min., 55°C for 30 sec., and 72°C for 30 sec. The PCR products were ligated into pCR^® ^II Topo^® ^vector (Invitrogen) and multiple sublcones sequenced. Sequences were aligned with Clustal X and analyzed in Meth Tools [39].

Two CpG islands (I and IV, Figure 2B) were not amenable to primer design due to the presence of repeats, and were thus analyzed by southern analyses. For CpG island I, 10 μg of genomic DNA was digested with *PstI *in conjunction with either *Msp I *or *Hpa II *to allow for the detection of a <1 kb band encompassing the boundary of this island. The probe was generated by amplification of DNA from forward (5': GCTTCATCGACTTCAGGCTG) and reverse (5': CTCAGTGGGGAGCTCTCCA) primers. For CpG island IV, 10 μg of genomic DNA from a Day 0 neonate was digested with *EcoR I *and *Xmn I *in conjunction with either *Msp I *or *Hpa II *to allow for the detection of a <1 kb band encompassing the boundary of this island. The probe was generated by amplification of DNA from forward (5': AAAGTCCGCAAGGGGATG) and reverse (5': CCCCCTTCAGTCCTCTCTCT) primers. In both assays, a shift, or lack thereof, in the size of the hybridizing fragments in the *HpaII *digests indicates the methylation status of the respective targeted CpGs.

Quantitative PCR (QPCR) of the region just upstream of the 5' noncoding exon (CpG island III) was performed using Biorad Sybr Green on Biorad iCycler machine as previously described [40]. Briefly, 2 μg genomic DNA isolated from non-treated and 5-azacytidine treated neonate cells was digested overnight at 37°C in separate reactions with either 40 Units of *Eco*RI (an enzyme targeting sites outside of the PCR target region), *Hpa*II (methylation sensitive) or *Msp*I (methylation insensitive) and subsequently cleaned using Microcon YM-100s according to the manufacturer's protocol. Primers spanning one CCGG (*Hpa*II/*Msp*I) targeting the CpG island III (see Additional file 1) amplified 50 ng of gDNA (undigested gDNA, *Eco*RI digested gDNA, *Hpa*II digested gDNA and *Msp*I digested gDNA) from either non-treated or 5-azacytidine treated cells with the following cycling conditions: 95°C for 15 sec, 63°C for 45 seconds in 40 total cycles. *Eco*RI digested DNA was analyzed by QPCR to verify there was no effect on amplification efficiency due to post-digestion processing (data not shown). *Msp*I digested DNA was analyzed by QPCR to verify there was complete digestion of *Hpa*II/*Msp*I sites regardless of methylation (no amplification detected, data not shown). The reference PCR product targeted the first coding exon and contained no restriction sites (see Additional file 1). Reactions were assembled in triplicate and normalized against the reference as in [41].

### Matrix attachment region fluorescence in situ hybridization (FISH)

Sequence obtained from BAC and lambda clones were input into the MAR-Wiz program v 1.5 [23] for analysis of potential matrix attachment regions. Halo preparation was performed by culturing fibroblast cells from *M. domestica *day 0 neonates as above. Nuclear halos were then prepared by the methods of [42] with the exception that dehydration following halo formation consisted of 10%, 30%, 70%, 90%, and 100% ethanol, and slides were fixed in methanol/acetic-acid (3:1) for 1 hour, then baked for 1 hour. The slide was treated with RNase A (0.1 mg/mL in 2 × SSC) for 1 hour and denatured in 70% formamide/2 × SSC at 70°C for 2 minutes prior to hybridization, then serially dehydrated at -20°C in 70%, 90%, and 100% ethanol and allowed to dry.

The *in situ *hybridization experiments were carried out with prepared halos using the 5 kb sublcone as above. Random priming with DIG-High Prime (Roche), DNA FISH probe preparation, hybridization and post-hybridization washes were performed as above for RNA-DNA FISH.

## Authors' contributions

BRL performed sequencing, sequencing analysis and MAR assays and wrote the manuscript. BRC performed sequencing and bisulphite and real-time PCR methylation assays. CJO and RJO performed RNA/DNA FISH. CMG performed methylation Southern analysis. JLV provided materials. II provided sequence analysis expertise. MJO and RJO conceived of and planned the experiments, obtained funding and wrote the manuscript. All authors participated in editing the manuscript.

## Supplementary Material

Additional file 1 CpG methylation analysis primer list and Region III sequence. Table of sequences of primers used in PCR and real-time PCR amplification of bisulphite treated genomic DNA, and text of Region III DNA sequence amplified by real-time PCR assay. Click here for file
