# Supplementary material for: Genomic imprinting of IGF2 in marsupials is methylation dependent
Source: BMC Genomics. 2008 May 2;9:205. doi: 10.1186/1471-2164-9-205 (PMC2386826; doi:10.1186/1471-2164-9-205)
Supplement: Additional file 1 — CpG methylation analysis primer list and Region III sequence. Table of sequences of primers used in PCR and real-time PCR amplification of bisulphite treated genomic DNA, and text of Region III DNA sequence amplified by real-time PCR assay. [file 1471-2164-9-205-S1.doc]

**Table 1.** CpG Methylation Analysis Primer List

| **Bisulfite PCR** | **Forward** | **Reverse** |
| --- | --- | --- |
| Region II | AGTTTGGGGTGAAGAGTTTGTATT | ACACCCAAAAAATACCAAAATAAC |
| Region V.I | TTAGGGTATTTGATGGGGGATT | CCACACACAAACTAAAAAATCTACC |
| Region V.ii | TGGGGATTTATTGTTGGGTTA | ACTAAACATACCATCCCCCAAAT |
| Region VI | ACTCTACTCTCTTCCTTCCT | GGGTAGAGTTTTTTTGGAAGTT |
|  |  |  |
| **Real Time PCR** |  |  |
| Region III | GCGGAGCGAGATAACCTTCT | TGCGAGTCCCCGACTACCTGC |
| Reference seq. | GAGCAGAGTCACCCTCCAAG | CTGTCCCTTCTCAGCACTCC |

**Region III (Figure 2A) Sequence.** Real Time PCR priming sites underlined; differentially methylated *Hpa*II/*Msp*I site highlighted in gray; 5’-untranslated exon in capital letters; all CpG residues in bold.

g**cg**gag**cg**agataaccttcttcc**cg**gccattgggt**cg**ggcctgcagccAGTGGTGACATCCAG**CG**AT**CG**GAG**CG**AGTTCTCCCAAAC**CG**TTATAAAAGGAGAGCTGGAGGG**CG**GATCTGTCTGTCTCCTCCTAGA**CG**GAGCAGgtagt**cg**gggact**cg**ca
